# COVID-19 Related Protocol Considerations and Modifications within a Rural, Community-Engaged Health Promotion Randomized Trial

**DOI:** 10.3390/mps6010005

**Published:** 2023-01-05

**Authors:** Rebecca A. Seguin-Fowler, Galen D. Eldridge, Meredith Graham, Sara C. Folta, Karla L. Hanson, Jay E. Maddock

**Affiliations:** 1 Institute for Advancing Health through Agriculture, Texas A&M AgriLife, College Station, TX 77845, USA; 2 Texas A&M AgriLife Research and Extension Center, Dallas, TX 75252, USA; 3 Friedman School of Nutrition Science and Policy, Tufts University, Medford, MA 02155, USA; 4 Department of Public and Ecosystem Health, Cornell University, Ithaca, NY 14853, USA; 5 School of Public Health, Texas A&M University, College Station, TX 77843, USA

**Keywords:** randomized trial, recruitment, COVID-19, rural

## Abstract

Rural communities are at higher risk for physical inactivity, poor dietary behaviors, and related chronic diseases and obesity. These disparities are largely driven by built environment, socioeconomic, and social factors. A community-based cluster randomized controlled trial of an intervention, the Change Club, aims to address some of these disparities via civic engagement for built environment change. Baseline data collection began in February 2020, only to be paused by the COVID-19 pandemic. In this context, the investigators evaluated multiple approaches for collecting data when the study resumed, focusing on Life’s Simple 7, and additional anthropometric, physiologic, and behavioral outcomes in rural and micropolitan (<50,000 population) communities in Texas and New York. Life’s Simple 7 includes fasting blood glucose, total cholesterol, blood pressure, weight, physical activity, diet, and smoking. Rigor and feasibility were considered across a variety of in-person versus at-home measurement options. After a comprehensive input from participants, partners, staff, researchers, and the funding liaison, the study team chose self-measurement and use of validated questionnaires/surveys to measure the Life’s Simple 7 components. This case provides an example of how a study team might adjust data collection protocol during unexpected and acute events while giving consideration to rigor, feasibility, stakeholder views, and participants’ health and safety.

## 1. Introduction

Chronic disease (e.g., diabetes, hypertension) and obesity rates are high across the United States and have increased over the past several decades [1,2,3]. Obesity has been linked to numerous poor health outcomes including cancer, heart disease, and all-cause mortality [4,5]. While chronic diseases and obesity are nationwide problems, rural communities are at higher risk for chronic diseases, obesity, and antecedent causes of physical inactivity and poor dietary behaviors [6,7,8]. Disparities in health behaviors related to chronic diseases and obesity in rural communities are driven, in part, by built environment (e.g., food deserts, low walkable communities), socioeconomic (e.g., poverty and low educational attainment), and social factors (e.g., cultural norms) [9,10,11,12].

To address these disparities, the investigators proposed a community-based cluster randomized controlled trial of an intervention called the Change Club (CC) in 12 paired rural communities in Texas and New York [13]. CC is a civic engagement for built environment change (CEBEC) intervention. Specifically, the CC intervention is comprised of convening a small group of residents and guiding them through a coordinated planning process to address built environment issues in their community while also enhancing social cohesion and connectedness not only within the small group, but also extending to the broader community [13]. The CC intervention is detailed in the protocol article [13]. Briefly, CC members are led through stepwise built environment change planning workshops, with the first set of modules focusing on building group rapport and identity and establishing group norms, and subsequent modules focusing on community assessment, issue identification, action planning, and selection from a menu of possible built environment changes that could be feasibly implemented in the community within the next six months (e.g., improving foods in restaurants or schools, improving parks or walking trails) [13]. The proposed trial protocol involved the recruitment of 2260 adults in three samples: CC members (*n* = 10–14 per community), friends and family of the CC members (*n* = 90–112 per community), and community members (*n* = 80–100 per community) [13]. Friends and family members and community members do not participate in the CEBEC curriculum but are included to assess whether there is an impact of the CEBEC intervention within the broader communities. Recruitment was planned to be led by extension educators who would also serve as CC facilitators. Planned recruitment efforts for all groups focused on community events and flyers posted throughout the community [13]. Secondary recruitment efforts included direct mailing of postcards, television and radio advertising, and social media postings [13].

The primary outcome is change in the American Heart Association’s Life’s Simple 7 composite cardiovascular health score [14], which includes three health risk factors (fasting blood glucose, total cholesterol, and blood pressure [BP]) and four health behaviors (weight, physical activity, diet, and smoking). Secondary outcomes included general health status [15]; healthy eating motivation [16], confidence [17,18], and social support [19]; and exercise attitudes [20], confidence [18], and social support [19,21] variables, as well as six variables at the community/collective level (social cohesion [22], social engagement [23], individual mobilization [24], civic engagement attitudes, civic engagement behaviors [25], and community health investment attitudes [26]) and six at the community environment level (neighborhood, safety, aesthetics, and walking environment [22] and availability of fresh fruit and vegetables, selection of stores, and healthy restaurant options [27]).

The study was funded by the National Cancer Institute in 2019 and after approximately one year of study preparations such as hiring staff, developing surveys and intervention materials, and gaining Institutional Review Board approval, study recruitment was launched in early 2020. During that same time period, the SARS-CoV-2 virus was rapidly emerging [28]. By January 30th, the World Health Organization declared the pandemic a global health emergency; by February 11, the disease was named COVID-19, and the first US death occurred on February 28 [28]. In early March 2020, rapid cancelation of events occurred, followed by business closings. Stay-at-home orders were enacted on March 20th in New York and on March 31st in Texas. 

Stay-at-home orders stopped in-person recruitment efforts, and the study team shifted to remote recruitment. The orders also resulted in a high level of uncertainty, since they were initially issued for only 30 days, after which it was unclear what would happen. Concurrently, and throughout the pandemic, trust in science fell, with only 29% of US adults having a great deal of confidence in medical scientists to have the public’s best interests in mind in February of 2022 compared to 45% in 2018 [29].

The project team continued with remote recruitment until it became apparent that in-person activities would not be resuming for the foreseeable future, and therefore objective measurements of health could not be collected. Study recruitment was then paused so that the team could reconsider how to conduct the study within the COVID-19-related restrictions and concomitant changes to the social and cultural context. The research team, National Institutes of Health program officer, and community partners discussed several options and decided that modifying the baseline data collection protocol was required.

Because of COVID-19-related restrictions and the cultural context, it was necessary to change the way some of the Life’s Simple 7 components would be measured. This paper describes the process of evaluating the options for collecting baseline data and selecting an approach that balanced both feasibility and rigor. We provide options that other researchers can consider when recruiting research participants and collecting data when in-person approaches may be challenging or impossible, and/or during times when trust in science/research is low/compromised. We discuss the tradeoffs that must be considered to meet enrollment targets and successfully adapt the proposed study. 

## 2. Methods

Because in-person measurement options were hindered due to COVID-19, we evaluated multiple approaches for collecting data, focusing on the primary outcome, Life’s Simple 7, and additional anthropometric, physiologic, and behavioral outcomes in the context of the cluster-randomized controlled trial in rural and micropolitan (<50,000 population) communities in the USA. Life’s Simple 7, aforementioned, includes the components of fasting blood glucose, total cholesterol, BP, weight, physical activity, diet, and smoking. Each component of Life’s Simple 7 is categorized as Poor (score of 0), Intermediate (score of 1), or Ideal (score of 2). Under all data collection approaches, research team staff, clinical staff, or study participants would be provided with standard protocols for measurement and recording of data. 

### 2.1. Measurement Options

#### 2.1.1. In-Person Measurement Options

In-person scenarios were given considerable consideration to try to preserve the rigor of the study, particularly of the outcomes measured via blood tests (blood glucose; hemoglobin A1c; and total, LDL, and HDL cholesterol), clinical outcomes (BP and heart rate), and anthropometric outcomes (height and body weight (to calculate Body Mass Index [BMI]); waist circumference). The original protocol involved measurement by the research team. It was intended that study staff would set up stations to have measurements taken on-site in each rural community. Blood would be collected by fingerstick and analyzed by point-of-care devices. Research staff would measure clinical and anthropometric outcomes. As an additional measure of diet beyond self-report, skin carotenoids would be assessed via Veggie Meter skin scan. A pre-specified number of people would be seen every 10 min for eight hours per day over a designated number of days of measurement in each community. Given that this approach was stymied by the beginning of the COVID-19 pandemic, which legally prohibited group gatherings in some areas, and gatherings could also generate fear that deterred participation, clinic-based setting options were considered as an alternative. In this scenario, participants would make individual appointments and clinic staff/phlebotomists would conduct venipuncture to collect vials of blood which would be processed in the clinic’s laboratory; results for all tests would be transmitted directly to the research team. Clinic staff would measure clinical and anthropometric outcomes. Skin carotenoids could not be measured in the clinic-based setting. 

#### 2.1.2. At-Home Measurement Options

As the pandemic was unrelenting and it became clear that potential study participants were resistant to in-person options, the study team considered at-home options and deliberated the resulting options (Table 1, column 2).

At-home measurement of blood glucose and lipids could be obtained through at-home blood microsampling. Each participant could be provided with a microsampling kit that included supplies and instructions for pricking a sterile finger with the lancet provided and filling several tiny tubes with blood. Participants would then package the blood tubes into a shipping container and send them to a laboratory that would transmit results directly to the research team (blood glucose; hemoglobin A1c; and total, HDL, and LDL cholesterol). In addition, providing all participants with a Bluetooth BP cuff that could transmit BP and heart rate measurements directly to the research team was considered for at-home measurement of BP and heart rate. 

Three options for at-home measurement of body weight were considered. All participants could be provided with a Bluetooth scale that could transmit body weight measurements directly to the research team. Alternatively, participants could be provided with a basic scale and a data entry sheet onto which they would record multiple body weight measurements and report the data online. Two basic-scale approaches were considered: providing all participants with a basic scale or providing a basic scale only to participants who had no scale of their own. For height and waist circumference, options considered were having participants measure and report their height and waist circumference using a tape measure provided to all participants or provided only to participants who reported needing/not having access to a tape measure. Self-assessed height, weight, and waist circumference protocols for this study were adapted from interviewer-assessed protocols used in the National Health and Nutrition Examination Survey [30] and previous study protocols [31,32].

Three approaches to measurement of physical activity were considered. First, physical activity could be measured using accelerometers loaned to participants for 7 days of wear. Devices would then be returned to the research team and data downloaded to provide valid and reliable estimates of MET-minutes/week; this approach has contamination considerations in the COVID-19 context. Thus, another set of approaches were considered: provide all participants with the same pedometer (less expensive than an accelerometer, so able to be provided rather than loaned) and a 7-day log onto which participants would record steps taken each day and report data online to attain estimates of participants’ mean daily steps, or to allow participants to use and track activity on an approved device they already owned.

#### 2.1.3. Questionnaires via Survey

Due to the pandemic, a more feasible approach for outcomes of interest was self-reporting. In terms of rigor, survey-based scales/instruments for Life’s Simple 7 components were available [33,34,35]. Survey options included questions about measurements, diagnosis, and medication use related to blood glucose, cholesterol, and BP [33,34,35]. These self-reported items could be used to determine Life’s Simple 7 component scores by classifying participants as having high (Poor), elevated or treated (Intermediate), or normal (Ideal) BP, total cholesterol, and blood glucose, as is standard protocol for Life’s Simple 7 scoring procedures. Survey questions about current height and weight could also be included to determine BMI.

Self-reported physical activity could be assessed using the Life’s Simple 7 physical activity item and/or using the International Physical Activity Questionnaire long form (IPAQ-long) [36,37,38] to attain MET-minutes/week. For diet, Life’s Simple 7 includes a standard eight diet-related items [33,39]. Five assess consumption of: (1) sugar-sweetened beverages (servings/day); (2) fish (servings/week); (3) fruit (cups/day); (4) vegetables (cups/day); and (5) whole grains (servings/day); and three additional questions queried whether participants (6) avoided eating prepacked/processed foods; (7) rarely ate out and sought lower sodium restaurant options; and (8) avoided adding extra salt when cooking [33,39,40,41]. In addition to the diet-related questions, 24 h dietary recalls could be collected via the Automated Self-Administered 24 h Dietary Assessment Tool (ASA24) [42].

Smoking is assessed by the Life’s Simple 7 criteria (never smoked or quit > 12 months ago; quit < 12 months ago; current smoker) [33,39].

### 2.2. Data Collection Scenarios

To organize options for protocol changes, the measurement choices were arrayed into data collection scenarios representing different combinations of in-person, at-home, and self-reported approaches (Table 2). The Research Team Measurement scenario represents the Change Club Study’s originally proposed plans for collection of outcome data from validated survey-based questionnaires supported by objective measurements of lipids and blood glucose from point-of-care blood analysis devices; measured height, weight, waist circumference, BP, and heart rate; carotenoids via skin scan; and total physical activity using accelerometers. These objective data would be collected in-person by research team staff at public events in the communities where participants resided. As this became infeasible, alternative scenarios were considered; these are described in Table 2.

### 2.3. Sample Size Calculations

The calculation of sample size targets for all three samples (140 CC members, 1120 family and friends, 1000 community residents) were detailed in the protocol article [13]. Briefly, targets are based upon the ability to detect significant differences in the Life’s Simple 7 total score between arms at the 24-month post-baseline time point, using a two-sided *t*-test, with 80% power and an alpha level of 0.05. We aimed to detect 24-month effects that were approximately equal in size to those observed in our prior intervention studies which had only 6-month follow-up (+0.7–0.8 units in the Life’s Simple 7 total score) [34,35]. All calculations assumed consistent cluster sizes across communities and accounted for attrition (10% for CC members, 20% for others) and the intraclass correlation within clusters (0.08 for friends and family members, 0.025 for others). Target sample sizes will yield an ability to detect change in the Life’s Simple 7 composite score of 1 unit among CC members, 0.4 units among family and friends, and 0.7 among community residents.

### 2.4. Cost Estimates

Cost estimates include contracts (e.g., ASA24, laboratory), equipment (e.g., Veggie Meters for carotenoid measurement, accelerometers), supplies (e.g., scales, pedometers), shipping, and travel necessary to implement data collection under each scenario. Costs are based on vendor quotes and online pricing information at the time each scenario was considered between 2019 and 2022. All prices have been adjusted into 2022 dollars using the annual Consumer Price Index [43]. Scenario costs do ***not*** include research staff time because it varies greatly for different organizations and pay rates across locations ***nor*** do they include participant compensation costs. Given the geographic distribution of study sites in rural locations across two states, we estimated that loaning accelerometers would require obtaining one accelerometer per 2.5 participants (thus, per participant cost includes 40% of the cost of purchasing an accelerometer). For the ‘Self-Measurement w/Equipment Provided As Needed Only’ scenario, we assumed that half of all participants would need equipment (pedometer, scale, tape measure) purchased and shipped to them.

### 2.5. Analysis

Total costs for each scenario were calculated and presented as a range (±25%). Rigor versus feasibility (including costs) and tradeoffs of each data collection scenario are discussed and compared.

## 3. Results

### 3.1. Rigor vs. Feasibility Considerations

Table 3 lists a comparison of benefits and drawbacks of each of the scenarios in terms of rigor and feasibility. In general, we considered objective measures to be more reliable than self-measured data, and self-measured data to be more reliable than self-reported data without any measurement activity. Scenarios described in the earlier rows generally had greater rigor (e.g., more objective measures, less social desirability bias) than later rows. Scenarios described in later rows were generally more feasible because they were less burdensome to participants (e.g., less time, no blood) than earlier rows. Some drawbacks, such as the lack of internet and cell service in rural areas, were pervasive but were bigger issues for data collection scenarios that relied more heavily on online reporting (e.g., Bluetooth devices, longer surveys).

### 3.2. Cost Comparisons

Costs varied depending upon the data collection approach used (Table 4). Self-Measurement w/Bluetooth Devices was the most expensive (USD 257-USD 428/participant), and Self-Measurement w/Equipment Provided As Needed Only (USD 20-USD 33/participant) and Surveys w/Self-Measurement Only if Equipment Already Available to Participant (USD 3-USD 5/participant) were the least expensive. The other three approaches had costs that were more similar to one another.

### 3.3. Selected Data Collection Approach

After considering rigor versus feasibility, all benefits and drawbacks, and the study budget and timeline, we chose the Self-Measurement w/Equipment Provided as Needed Only scenario for data collection. In this case, participants were instructed to use equipment they had on hand, and if they did not have the basic items needed, they were mailed to them and kept by the participant for future use. The Self-Measurement w/Equipment Provided as Needed Only scenario required no CC participants to travel or get blood drawn, but still collected data comparable to some national surveys [44]. For some CC participants, burden was further reduced because they could use equipment they already owned (e.g., scale, fitness tracker), and researchers will not have to replace lost equipment in future years.

We made two additional adaptations to the protocol—one to enhance feasibility and the other to strengthen rigor. First, given employment and time stressors among adults in these underserved rural populations, we increased participant compensation from USD 50/year to USD 75/year for complete data. Second, we added items to the questionnaire that enabled us to calculate the 2018 World Cancer Research Fund/American Institute for Cancer Research (WCRF/AICR) cancer prevention score [45]. Although the 2018 WCRF/AICR score is correlated with cancer incidence and mortality [46,47,48,49,50,51] and is recommended for use in intervention studies as a standardized cancer prevention score [45], published data from intervention studies using the score as designed are still very limited. We added diet-related survey questions adapted from the National Health and Nutrition Examination Survey (NHANES) dietary screener questionnaire [19] and the Beverage and Snack Questionnaire [20] to assess: (1) fiber intake (g/day); (2) frequency of consuming ultra-processed foods (times/month); and (3) red and processed meat consumption (g/week) [19,21]; and (4) two questions adapted from the Alcohol Use Disorders Screening Test [22] to assess alcohol consumption (drinks/day). The 2018 WCRF/AICR score will allow us to broaden conclusions about the public health impact of the CC intervention.

## 4. Discussion

As originally proposed, five of the Life’s Simple 7 components would have been measured onsite by the research team: fasting blood glucose and total cholesterol via fingerstick and point-of-care device; BP via BP monitor; weight via digital body weight scale; and diet (fruit and vegetable component) via skin carotenoid measurement using Veggie Meter skin scans. For physical activity, accelerometers were to be loaned to participants and returned at in-person meetings. The original protocol also included 24-h recall and validated questionnaires to measure Life’s Simple 7 diet components. Smoking was to be assessed via questionnaire. The selected data collection approach decision within the COVID-19 context, considering rigor and feasibility, included three Life’s Simple 7 components measured by the participant at home either using equipment on hand or equipment mailed by the study team if equipment was not already available at home. All other measures were measured via questionnaire.

Studies propose the optimal design that is maximally rigorous to address the research questions as well as feasible from financial and practical perspectives. The COVID-19 pandemic brought forth numerous new contextual considerations including legal restrictions and public health recommendations that continually evolved, as well as stress and strain on people’s time and capacity. For example, in the rural locations in this study, considerations included balancing childcare and home schooling with fulltime remote work and often with poor and unreliable internet; inability to gather or fear about gathering; lack of trust and political polarization related to public health; and clinic closures, low staff, limited clinic hours, and thus often further distances for people already facing resource constraints to travel to clinics. Thus, we optimized the study to collect all data for the primary outcome plus the 2018 WCRF/AICR score (but via self-report/measurement rather than in-person measurement, which was no longer feasible) and increased participant compensation given the new realities and time burdens within our rural communities.

At the time of the study proposal, the planned Research Team Measurement scenario for recruitment, enrollment, and baseline data collection was completely feasible, and based on our experience in other rural settings and studies [31,34,35], would have been likely to attract an unbiased sample (i.e., participants a mix of healthy/less healthy and motivated/less motivated to change). With the onset of COVID-19 restrictions and recommendations, the Research Team Measurement scenario become unfeasible due to the illegality and health risks of gathering in large groups. The scenario which would have resulted in obtaining all of the planned objective measures with the exception of skin carotenoids, the Clinic-Based Measurement scenario, involved asking participants to travel to clinics, which, in these rural areas, would have involved travel of up to 60 miles, as well as venipuncture, both of which may be unacceptable for large numbers of participants. The Clinic-Based Measurement scenario would have attracted highly motivated participants, willing to take time to travel, enter clinics at a time when people may have been trying to avoid places where people were sick, and have venipuncture performed. The Self-Measurement w/Bluetooth Devices scenario would have avoided self-report bias by directly reporting some measurements to the research team, but was the scenario with the highest total cost, and lack of internet in rural areas would have greatly hindered Bluetooth device use. (Note that Bluetooth itself does not require internet but conveying the results from the Bluetooth device to the research team does require internet access.)

Based on feedback from educators and residents, any increase in participant burden (e.g., requiring driving long distances to clinics, venipuncture) would increase the likelihood that only highly motivated (likely already healthy individuals) would enroll and thus bias samples. Lowering participant burden would increase the chance of enrolling participants who could benefit more from the intervention (i.e., less healthy) and who were more representative of the residents of the communities. Considering all these benefits and drawbacks, the Self-Measurement w/Equipment Provided as Needed Only scenario was selected. This scenario is similar to how data are gathered for nationally representative datasets by the US government and the American Heart Association’s monitoring of Life’s Simple 7. For example, NHANES, a national program designed to assess health and nutritional status in the US, uses both self-report and objective measurements [44]. The American Heart Association’s My Life Check is a health assessment and improvement tool that uses Life’s Simple 7 (recently updated to Life’s Essential 8 to include sleep) to help people work toward improving their health [52]; this tool uses self-report data. Furthermore, we can conduct sensitivity analyses that adjust self-report for social desirability bias by using correction calculations derived from other large studies (e.g., for weight in the Women’s Health Initiative [53]).

Based on our experiences, we have multiple recommendations for feasibility, rigor, and protocol changes. If protocol changes seem as though they will be needed, implement them early and make sure to get buy-in across all stakeholders including community partners, educators/facilitators, and potential participants. Consider adequate compensation from the proposal stage and limit data collection to the essential measures to reduce long-term follow-up and participant fatigue. Consider ways to provide people with equipment that limits shipping and insurance costs, as well as participants’ need to drive long distances (e.g., grocery store, “site,” and school pickups). Consider hybrid data collection methods; it is not always possible to get the data in exactly the same way from all participants in a pragmatic study. Be flexible across self-reported, self-measured, and research- or clinic-based data collection methods while still being able to address core research questions. Use analytic strategies to check robustness of findings controlling for different methods of data collection across participants in a single study.

Local partners are critical for reflecting what protocol change considerations may or may not work well within their community and/or a particular population subgroup (e.g., moms with children, older adults). Valid, reliable, low participant burden technologies, particularly passive ones, for diet and physical activity data collection at the individual level are urgently needed.

## 5. Conclusions

There is often tension between rigor and feasibility in selecting data collection approaches for human research studies. Unexpected and acute events, such as the COVID-19 pandemic, force reconsideration of a study protocol with these two competing factors at the forefront. This case provides an example of how a study team might weigh these factors, as well as a process that included considerations from the funder as well as community voices. While new technologies may help reduce the conflict between these two factors in the future, it is important to discuss options transparently and have solid processes for decision-making.

## Figures and Tables

**Table 1:** Methods of data collection via in-person and at-home measurements, and self-reported options.

Outcomes	In-Person Measurement Options	At-Home Measurement Options	Self-Reported Options
Blood Sugar	Measured by research team with point of care device Blood draw at clinic/office	- Blood microsampling	Questions via survey
Lipids			
Blood Pressure	3. Measured by research team 4. Measured at clinic/office	- Provide Bluetooth BP cuff to all participants	Questions via survey
Heart Rate			None
Body Mass Index	5. Measured by research team 6. Measured at clinic/office	7. Provide Bluetooth scale to all participants 8. Provide basic scale to all participants 9. Provide basic scale only to participants who do not have one	Questions via survey
Waist Circumference	10. Measured by research team 11. Measured at clinic/office	12. Provide tape measure to all participants 13. Provide tape measure only to participants who do not have one	Questions via survey
Diet	Skin carotenoids measured by research team with Veggie Meter		24-h recall (ASA-24) and Questionnaires via survey
Physical Activity		14. Loan accelerometers to all participants 15. Provide pedometers to all participants 16. Provide pedometers to participants who do not have pedometer or activity tracker	IPAQ-long via survey

**Table 2:** Six data collection scenarios.

Scenario
**Research Team Measurement (original protocol) ** Questionnaires via survey 24-h recall Accelerometers loaned to all Measurements, point-of-care blood work, and skin carotenoid scans by research staff
**Clinic-Based Measurement ** Questionnaires via survey 24-h recall Accelerometers loaned to all Measurements and blood draw at clinic/office **No skin carotenoid scan**
**Self-Measurement w/Bluetooth Devices ** Questionnaires via survey 24-h recall Pedometer to all At-home measurements with Bluetooth devices (scale and BP cuff) to all Self-measurement with tape measure to all Blood microsampling at home
**Self-Measurement w/Standardized Basic Equipment Always Provided ** Questionnaires via survey 24-h recall Pedometer to all Self-measurement with basic equipment (scale, tape measure, and BP cuff) to all Blood microsampling at home
**Self-Measurement w/Equipment Provided As Needed Only ** Questionnaires via survey, expanded to include questions on blood glucose, cholesterol, and BP 24-h recall Pedometer automatically provided to those who do not have pedometer or activity tracker Self-measurement basic equipment (scale and/or tape measure) provided to those who do not have one ***No measure of heart rate***
**Surveys w/Self-Measurement Only if Equipment Already Available to Participant** Questionnaires via survey, expanded to include questions on body size, BP, blood glucose, and cholesterol; participants instructed to self-measure when equipment or information could be obtained.

**Table 3:** Benefits and drawbacks of each approach.

Scenario	Benefits	Drawbacks
**Research Team** **Measurement** **(original protocol)**	Maximize objective measurements, including skin carotenoids a valid biomarker for fruit and vegetable intake Research team measurements have high reliability and validity Participants may be comfortable with familiar research team staff Accelerometry provides most valid assessment of total physical activity As proposed, passed peer-reviewed standards for scientific rigor and reproducibility	Participants required to travel to data collection events; high level of reluctance in pandemic In-person data collection time slots are limited to several days Group data collection events may spread disease Participants required to travel to return accelerometer Point-of-care blood tests require fingerstick, which some find unpleasant Lack of internet and cell service in rural areas may hinder survey completion
**Clinic-based Measurement**	Maximize objective measurements, except for skin carotenoids Clinic measurements have high reliability and validity Participants may be comfortable with medical personnel taking blood at clinic Accelerometry provides most valid assessment of total physical activity For feasibility, participants had more flexibility in scheduling appointment at clinics	Limited number of clinics near rural communities required participants to travel up to 60 miles Reluctance to increase exposure to virus with non-essential clinic visits Participants required to travel to return accelerometer Laboratory blood tests require venous blood draw, which some find unpleasant Lack of internet and cell service in rural areas may hinder survey completion
**Self-Measurement w/Bluetooth Devices**	No travel required Measurements can be completed at the participant’s convenience Standardized equipment is reliable Bluetooth devices report directly, avoiding self-report bias	Lack of internet in rural areas may hinder both Bluetooth-device use and survey completion Pedometers provide only step counts, not total physical activity Participants reluctant to draw own blood, even with microsampling
**Self-Measurement w/Standardized Basic Equipment Always Provided**	No travel required Measurements can be completed at the participant’s convenience Standardized equipment is reliable	Self-measured and reported data may be subject to social desirability bias Equipment could be lost or given to participants that do not remain in the study Pedometers provide only step counts, not total physical activity Lack of internet and cell service in rural areas may hinder survey completion Heart rate not recorded
**Self-Measurement w/Equipment Provided As Needed Only**	No travel required No blood required More similar to national datasets and to how the American Heart Association has individuals complete the Life’s Simple 7 Some participants do not have to wait for equipment to arrive Less equipment to be lost and need to be replaced in future years Participant burden reduced (for some) by incorporating fitness tracker physical activity data	Survey is longer and lack of internet service may be bigger problem More outcomes rely only on self-report and are subject to recall and social desirability biases Using available equipment reduces measurement reliability Categorization of participants by risk for chronic diseases less reliable than lab values Heart rate not recorded
**Surveys w/Self-Measurement Only if Equipment Already Available to** **Participant**	No equipment required No travel required No blood required More similar to national datasets and to how the American Heart Association has individuals complete the Life’s Simple 7 Shorter wait time for compensation	Survey is longer and lack of internet service may be bigger problem Outcomes rely only on self-report/self-measurement and are subject to recall and social desirability biases Categorization of participants by risk for chronic diseases less reliable than lab values Heart rate not recorded

**Table 4:** Per participant data collection costs under each scenario.

Scenario	Cost/Participant
Research Team Measurement	USD 162–USD 270
Clinic-Based Measurement	USD 126–USD 210
Self-Measurement w/Bluetooth Devices	USD 257–USD 428
Self-Measurement w/Standardized Basic Equipment Always Provided	USD 141–USD 235
Self-Measurement w/Equipment Provided As Needed Only	USD 20–USD 33
Surveys w/Self-measurement Only if Equipment Already Available to Participant	USD 3–USD 5

## Data Availability

Not applicable.
